# Integrated single-cell and bulk characterization of cuproptosis key regulator PDHB and association with tumor microenvironment infiltration in clear cell renal cell carcinoma

**DOI:** 10.3389/fimmu.2023.1132661

**Published:** 2023-06-07

**Authors:** Jiajin Wu, Songbo Wang, Yiyang Liu, Tongtong Zhang, Xiaoyi Wang, Chenkui Miao

**Affiliations:** ^1^ Department of Urology, The First Affiliated Hospital of Nanjing Medical University, Nanjing, China; ^2^ Department of Urology surgery, Shuguang Hospital Affiliated to Shanghai University of Traditional Chinese Medicine, Shanghai, China; ^3^ Core Facility Center, the First Affiliated Hospital of Nanjing Medical University/Jiangsu Province Hospital, Nanjing, China

**Keywords:** cuproptosis, PDHB, tumor microenvironment, sunitinib, renal clear cell carcinoma, single-cell RNA-seq

## Abstract

**Background:**

Renal clear cell carcinoma (ccRCC) is one of the most prevalent cancers worldwide. Accumulating evidence revealed that copper-induced cell death played a vital role in various tumors. However, the underlying mechanism of cuproptosis with molecular heterogeneity and tumor microenvironment (TME) in ccRCC remains to be elucidated. The present study aimed to discover the biological function of cuproptosis regulators with the potential to guide clinical therapy.

**Methods:**

Using Single-cell RNA-seq, bulk transcriptome and other multi-omics datasets, we identify essential cuproptosis-related hub gene PDHB for further study. The dysregulation of PDHB in ccRCC was characterized, together with survival outcomes, pathway enrichment and immune infiltration among tumor microenvironments. The functional significance and clinical association of PDHB was validated with loss of function experiments and surgical removal specimens.

**Results:**

PDHB mRNA and protein expression level was significantly downregulated in ccRCC tissues compared with normal and paired normal tissues. Clinicopathological parameters and tissue microarray (TMA) indicated that PDHB was identified as a prognostic factor for survival outcomes among ccRCC patients. Additionally, low PDHB was negatively correlated with Treg cells, indicating an immunosuppressive microenvironment. Mechanistically, knockdown PDHB appeared to promote the RCC cells proliferation, migration, and invasion potentials. Subsequent studies showed that copper-induced cell death activation could overcome sunitinib resistance in RCC cells.

**Conclusion:**

This research illustrated a cuproptosis-related hub gene PDHB which could serve as a potential prognostic marker and provide therapeutic benefits for clinical treatment of ccRCC patients.

## Introduction

Renal cell carcinoma was one of top ten malignant cancer subtypes worldwide, which approximately affected 79000 individuals in 2022 (1, 2). There are several subvariants of RCC, including clear cell RCC (ccRCC), papillary RCC (pRCC), and chromophobe RCC (chRCC) (3). Among them, ccRCC was the most common pathological subtype accounting for 70% patients (4). Although increasing diagnosis strategies in early stage and progressive development of surgical management help to improve the level of efficacy, around 1/3 cases will eventually present local recurrence or distant metastasis (5, 6). Targeted drugs, including vascular endothelial growth factor (VEGF) inhibitors and mammalian targets of mTOR pathway, have been widely used as first-line treatment for metastatic renal cell carcinoma, which exhibited curative effectiveness (7–9). However, intolerance to TKIs treatment and poor drug response was still a major challenge (10–12). Even more, numerous patients developed unavoidable resistance towards TKIs like sunitinib and typically progress over time (13–17). Therefore, exploring potential drug targets and combination therapeutic strategies are increasingly crucial for optimizing survival outcomes.

Copper is one of essential metal nutrient for human body within the appropriate concentration range (18). Excessive accumulation of copper could trigger cell death and disease, such as Wilson’s disease and Menke’s disease (19). Accumulated evidence has proved that copper can induce apoptosis and autophagy through multiple mechanisms, including reaction to oxidative stress and proteasome inhibition (20, 21). According to recent research, Todd et al. investigated that copper ionophores induced a distinct form of regulated cell death (22). In contrast to traditional cell death pathways that we were familiar with, copper ionophore–induced cell death is nonapoptotic, non-ferroptotic, and non-necroptotic, and is dependent on copper and mitochondrial respiration (23). Performing genomic-wide CRISPR-Cas9 screens, several genes were filtered that could protect against copper-induced cell killing (24). Mechanistically, researchers reported that abnormal copper promotes the aggregation of lipoylated proteins and links mitochondrial metabolism to copper-dependent death (24). Elesclomol is a copper-binding compound, which could induce ROS, apoptosis and cuproptosis, which is characterized as a novel copper-dependent cell death mechanism (18, 20, 22, 24, 25). Nevertheless, the particular function of cuproptosis in tumor microenvironment during the development and progression of ccRCC remained to be further elucidated.

In this research, we utilized multiple algorithms to identify essential cuproptosis-related hub gene PDHB methodologically. The dysregulation of PDHB in ccRCC was associated with survival outcomes, pathway activation and immune infiltration among tumor microenvironments. The functional significance and clinical association of PDHB was validated with loss of function experiments and clinical samples. Collectively, we provided new insights and discovered potential mechanisms for using copper ionophores to overwhelm ccRCC.

## Materials and methods

### Data collection and processing

For single-cell RNA-seq, we collected three datasets of ccRCC patients and normal kidney tissues were downloaded from GEO database, including GSE131685, GSE152938 and GSE156632 (26, 27). We integrated all these scRNA through “Harmony” algorithm and gathered total 9 ccRCC and 9 normal kidney samples (28, 29). The standard workflow of cell clustering in Seurat was utilized to identify distinct groups of cells based on the integrated data. In brief, PCA was performed on the scaled data, and the top 20 PCs were used for graph-based clustering to identify cell clusters. Cluster marker genes were identified using “FindAllMarkers” function in Seurat (https://satijalab.org/seurat/) based on the “RNA” assay (30, 31). Next, respective reduction of cell clustering, including UMAP and PCA were performed, and cell cluster was obtained through the UMAP method. Finally, we used the “singleR” package to get the cell type for cell population annotation (32).

Then, for bulk RNA-seq, we integrated the normalized RNA-seq profiles (TPM), matched clinical characteristics and survival information of ccRCC samples and normal kidney samples from The Cancer Genome Atlas (TCGA, https://portal.gdc.cancer.gov) and GTEx database were downloaded (33). Meanwhile, GEO dataset GSE40435 was also applied to analyze (34). Differentially expressed cuproptosis regulators between tumor and normal tissue samples were screened out with the Wilcoxon test and “Limma” R package. Additionally, proteogenomic expression profiles of ccRCC patients was downloaded and pre-processed from CPTAC database and the **supplemental materials** of Ding’s research (35, 36).

Genes or proteins with false discovery rate (FDR) adjusted *P* < 0.05 and | log_2_FC (fold-change) | > 0.5 were considered as DEGs.

### Biological functional enrichment analysis

We conducted Gene Ontology (GO) enrichment analysis, which including biological process (BP), cellular components (CC), molecular function (MF), together with Kyoto Encyclopedia of Genes and Genomes (KEGG) pathways to explore the biological functions and underlying signaling pathways. Subsequently, gene set enrichment analysis (GSEA) and gene set variation analysis (GSVA) were performed to evaluate the pathways enriched among “h.all.v7.5.1.symbols.gmt” and “c2.cp.kegg.v7.4.symbols.gmt” gene sets from the molecular signature database (37, 38). We applied “AddModuleScore” algorithm to calculate the copper-induced cell death score in our scRNA datasets.

### Evaluating extent of immune cell infiltration abundance in tumor immune microenvironment

To exhibit the comprehensive landscape of immune cell infiltration in different subgroups, we conducted several deconvolution algorithm algorithms, including XCELL (39, 40), TIMER (41, 42), QUANTISEQ (43, 44), MCPCOUNT (45), EPIC (46), CIBERSORT (42, 47) and CIBERSORT-ABS (48) to estimate the subpopulations of immunity infiltration scores. Differences between two risk groups were analyzed by the Wilcoxon signed-rank test and the results were obtained according to p-value< 0.05. Subsequently, we used correlation analysis when exploring the relationship between the risk score and immune infiltrated cells.

### Clinical samples collection, tissue microarray and immunohistochemistry

Renal clear cell carcinoma and adjacent noncancerous renal samples were obtained by radical nephrectomy from the First Affiliated Hospital of Nanjing Medical University (Jiangsu Province Hospital) between 2005 and 2018. All diagnoses were confirmed by senior pathologists independently. Informed consent from all patients was acquired in the study. The study design and protocol were approved by the ethics committee of the First Affiliated Hospital of Nanjing Medical University (Jiangsu Province Hospital). IHC assays were performed as previously described (49, 50). Briefly, the primary antibody was diluted as follows: anti-PDHB (1:100, Abcam, USA).

### Cell culture, cell proliferation, migration, and invasion assays

RCC cell lines (786-O, 769-P, A498, Caki-1) and human renal tubular epithelial cell line (HK-2) were purchased from ATCC and cultured in RPMI 1640 (786-O, 769-P), McCoy’s 5A (Caki-1), DMEM (A498) and DMEM/F12 (HK-2) (Gibco, Thermo Fisher Scientific, USA) containing 10% fetal bovine serum (FBS) and 1% penicillin/streptomycin (Gibco, Thermo Fisher Scientific, USA). Small interfering RNA targeting PDHB (si-PDHB), and negative control (shNC) were constructed and transfected. Elesclomol (Selleck, China), a specific copper-induced cell death activator, was also applied. Cells were transfected with si-PDHB and si-NC using Lipofectamine 3000 (Invitrogen, Thermo Fisher Scientific, USA).

Pretreated RCC cells were counted and seeded into a 96-well plate at a density of 1.0x10^3^ cells/well. Cell proliferation was detected after 24h, 48h, 72h, and 96h using the CCK-8 Cell Counting Kit (Vazyme, China). The absorbance was measured at 450 nm with a microplate reader following incubation at 37°C for 1h according to the manufacturer’s protocols. For the colony formation assay, pretreated cells were seeded into 6-well plates (1000 cells/well). The cells were incubated for 10 days. Colonies were fixed in 4% paraformaldehyde for 20 min, washed with PBS twice, and stained with 0.1% crystal violet for further analysis.

For transwell cell migration and invasion assay, 1.5×10^5^ cells RCC cells were seeded into the 8μm PET membranes 24-well Transwell (Corning, USA) upper chambers with serum-free medium for the migration assays. Medium containing 15% FBS was added to the bottom chamber. After incubation at 37°C for 24 h, the cells were fixed in 4% paraformaldehyde for 20 min and stained with 0.1% crystal violet for 20 min. Cells were captured on a microscope in five randomly selected fields and repeated three times.

### RNA isolation and quantitative real‐time PCR assay

Total RNA was isolated using Trizol (Invitrogen, Thermo Fisher Scientific, USA). HiScript III All-in-one RT SuperMix (Vazyme, China) was used for cDNA synthesis. qRT-PCR was performed with SYBR qPCR Master Mix (Vazyme, China) using StepOne Plus (Applied Biosystems, USA) and LightCycler 480 PCR instrument (Roche Diagnostics, Switzerland) according to the manufacturer’s instructions. The primers and siRNA Oligo used were listed in **Table S1**.

### Tumor *in vivo* assays

All mice involved in this research were approved by the Institutional Animal Care and Use Committee (IACUC) of Nanjing Medical University. Briefly, total 2.5 × 10^7^ 786-O cells with knockdown-PDHB (shPDHB) and negative control cells were collected and suspended with PBS and Matrigel (1:1, Corning, USA), then subcutaneously injected into 4-week-old female BALB/c nude mice. The formula of tumor volume was calculated as follows: Tumor volume= (length*width)^2^/2.

### Statistical analysis

All analyses were performed using GraphPad Prism software and R 4.2.2. All statistical tests were two-sided, and P-value <0.05 was considered statistically significant unless otherwise noted. Continuous variables in normal distribution were between-group compared through the independent Student’s two-tailed t-test, while continuous variables in skewed distribution through the Mann-Whitney U test. Spearman order correlation analysis was used to determine the relationship between different subgroups. The differences in clinical outcomes were calculated with the Log-rank test through the Kaplan-Meier method. The univariate regression model was constructed to analyze the effect of each variable on the survival. All experiments were repeated independently three times. Data are shown as the mean ± standard deviation (SD). The P-value < 0.05 was considered to be statistically significant.

## Results

### Dysregulation and survival outcomes of cuproptosis regulators across pan-cancer types

**Figure 1:**
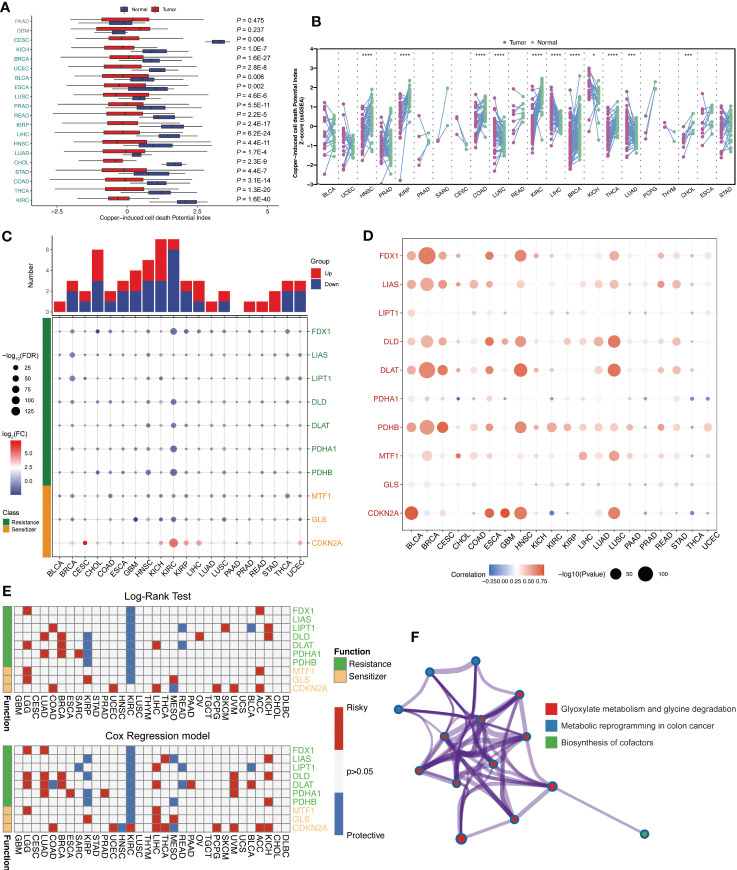
Landscape of expression level and survival outcomes of across human pan-cancer. **(A)** The cuproptosis score based on the gene expression from the TCGA database calculated by ssGSEA algorithm. **(B)** Cuproptosis score was significantly downregulated in multiple cancers. **(C)** The expression patterns of these cuproptosis regulators among TCGA pan-cancer dataset. **(D)** Somatic copy number alterations (SCNA) frequency of these cuproptosis regulators. **(E)** The association between cuproptosis resistances and overall survival outcomes. **(F)** Functional enrichment analysis of these cuproptosis regulators genes. *: p<0.05; ***: p<0.001; ****: p<0.0001.

Firstly, after summary copper-induced cell death regulators by genomic-wide CRISPR-Cas9 loss of function screening results, total 10 copper-induced cell death regulators were categorized into two groups: cuproptosis resistances (FDX1, LIAS, LIPT1, DLD, DLAT, PDHA1, and PDHB) and cuproptosis sensitizers (MTF1, GLS, and CDKN2A). For investigating the activity of copper-induced cell death across human cancers, single sample gene set enrichment (ssGSEA) algorithm was applied to calculate the cuproptosis score (CPS) based on the gene expression from the TCGA database. We found that CPS is significantly downregulated in the majority cancers (**Figure 1A**). Consistently, CPS was dramatically decreased in paired samples among human cancers (**Figure 1B**). These results confirmed that CPS based on different approaches or cancer subtypes is robust. We observed the aberrant expression patterns of these cuproptosis regulators (**Figure 1C**). Meanwhile, we visualized somatic copy number alterations (SCNA) frequency and the expression of these cuproptosis regulators in TCGA pan-cancer cohort (**Figure 1D**). Additionally, we analyzed the association between these cuproptosis regulators and overall survival outcomes by log-rank test and Cox regression. Interestingly, high expression of these cuproptosis resistances revealed favorable overall survival (**Figure 1E**). Functional enrichment further demonstrated that these intersecting genes were mainly enriched in glyoxylate metabolism and glycine degradation, metabolic reprogramming, and biosynthesis of cofactors (**Figure 1F**). These suggested that transcriptional alternations and genetic mutation of cuproptosis regulators are probably the underlying mechanisms leading to perturbations in copper-induced cell death.

### Distinct landscape of cuproptosis regulator genes in ccRCC

**Figure 2:**
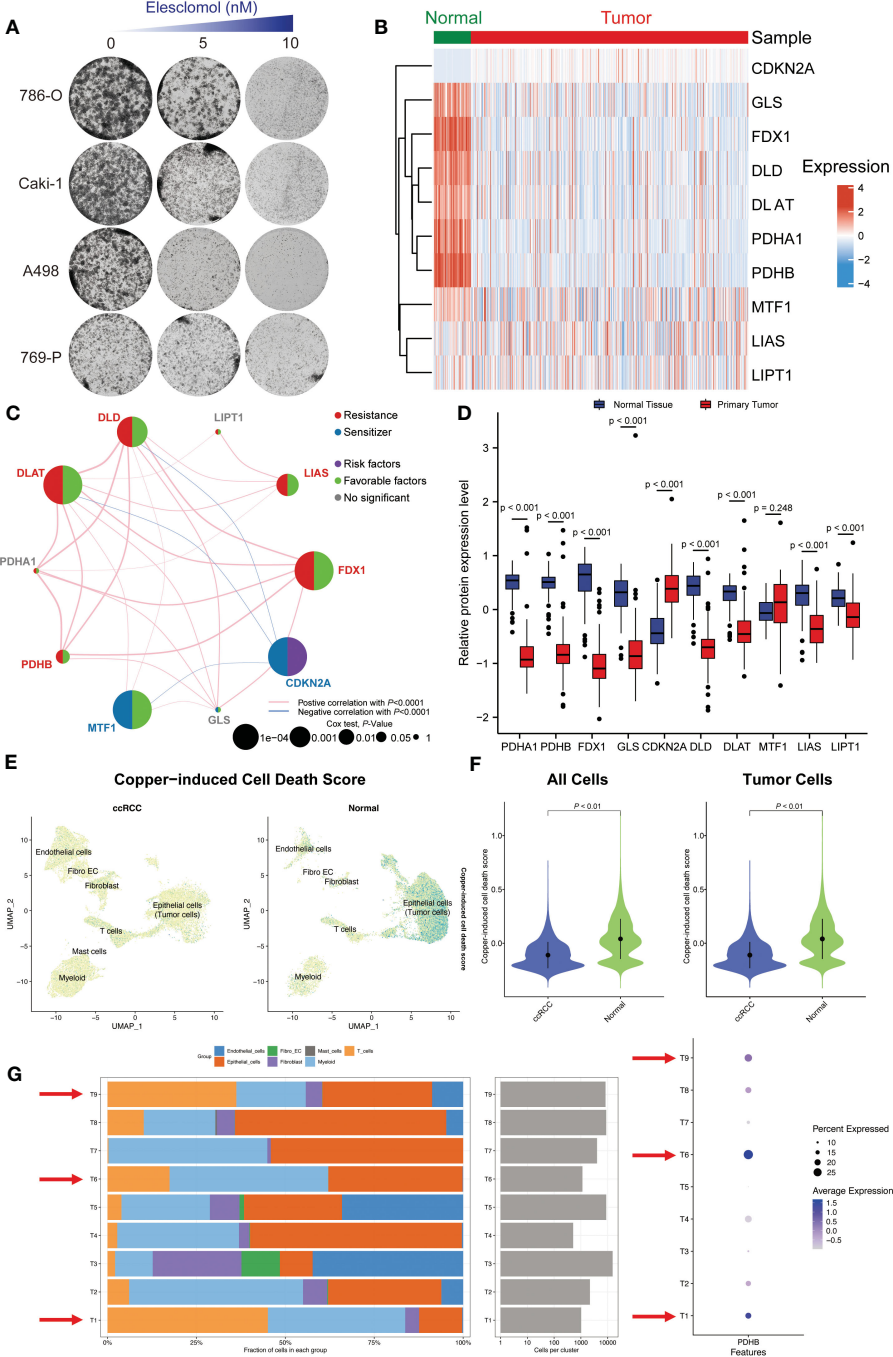
Single-cell and bulk RNA-seq revealed the distinct landscape of cuproptosis regulator genes in ccRCC. **(A)** The cell viability of 786-O, Caki-1, A498 and 769-P cells decreased significantly after cuproptosis activator (elesclomol) treatment. **(B)** The expression pattern of these cuproptosis-related genes in ccRCC and adjacent normal tissues. **(C)** Correlation analysis of these cuproptosis-related genes. **(D)** The protein expression level of these cuproptosis regulators in CPTAC-ccRCC database. **(E)** Single-cell RNA-seq illustrated the distribution of copper-induced cell death scores by “AddModuleScore” algorithm. **(F)** Copper-induced cell death signature was significantly down-regulated in ccRCC tumors tissues compared with normal samples both in all cells and tumor cells. **(G)** The cell proportion among different patients and correlations of PDHB expression level.

Based on above results, we discovered that ccRCC tissues had significantly lower copper-induced cell death index compared to adjacent normal tissues. Moreover, these cuproptosis regulators are abnormally expressed and associate with clinical outcome in ccRCC. Accordingly, we chose ccRCC for further research. First, we applied renal cancer cell lines to elesclomol exposure, a specific copper-dependent cell death activator. The cell viability of 786-O, Caki-1, A498 and 769-P cells decreased significantly after elesclomol treatment, which exhibited a concentration-dependent effect (**Figure 2A**). To further investigate the expression pattern of these cuproptosis-related genes in ccRCC, we quired TCGA-KIRC cohort to compare the transcriptional alternations, which was illustrated in a heatmap (**Figure 2B**). In addition, we analyzed the correlation between the expression of different genes and survival outcomes in ccRCC patients, which revealed strong associations. Among them, FDX1, DLAT, DLD, LIAS and PDHB showed positive correlation in cuproptosis resistances subgroup (**Figure 2C**). We also observed PDHA1, PDHB, FDX1, GLS, DLD, DLAT, LIAS and LIPT1 were down-regulated in ccRCC tumor tissues, while CDKN2A exhibited higher protein expression among tumor samples in CPTAC-ccRCC database (**Figure 2D**).

### Single-cell RNA sequencing revealed the distribution and expression of copper-induced cell death in ccRCC

In consideration of the heterogeneity of ccRCC, we applied single-cell RNA sequence (GSE) for further validation. Firstly, we after using “Harmony” to remove batch effects, we gathered a total of 33 clusters by UMAP algorithm (**Figure S1A**). Then, we explored the distribution of copper-induced cell death scores by single-cell signature scorer and found that overwhelming majority copper-induced cell death signature was enriched in normal samples compared with ccRCC tumors by “AddModuleScore” algorithm (*P*<0.001; **Figures 2E, F**). Marker genes between each cluster were calculated and illustrated in **Figure S1C**. ccRCC and normal kidney samples could mainly be divided into epithelial cells (Malignant tumor cells), endothelial cells, Myeloid cells, Mast cells, T cells, Fibroblast cells and Fibroblast_Endothelial_like cells (**Figures S1B**, **S2E**). Finally, we tried to explore the exact distribution of cuproptosis regulator genes in ccRCC tissues. Specifically, we conducted single-cell analysis to demonstrate and validate the detail changes of immune composition alternations and found that higher proportion of T and Myeloid cells among patients with high expression of PDHB, especially in patient sample T1, T6 and T9. In contrast, among low PDHB expression patients, we observed lower immune cell infiltration, such as T cells and myeloid cells **(** **Figure 2G**). The results showed that cuproptosis regulator genes were mainly concentrated in hepatocytes and epithelial cells, indicating their vital role in immune cell infiltration among tumor microenvironments.

### Identification of essential cuproptosis regulator PDHB in ccRCC

**Figure 3:**
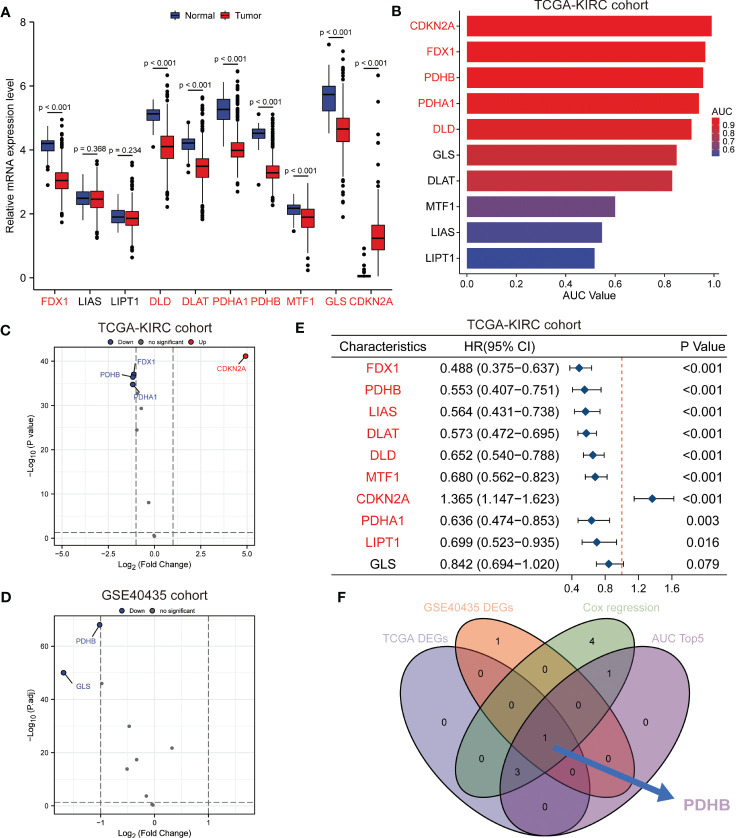
Identification of essential cuproptosis regulator PDHB in ccRCC. **(A)** The expression level of these cuproptosis regulator candidates in TCGA-ccRCC cohort. **(B)** The prognostic value of these cuproptosis-related genes calculated by the ROC curve and AUC value in TCGA-ccRCC cohort. **(C, D)** Differently expression analysis of cuproptosis regulators among TCGA-KIRC cohort **(C)** and two regulators among GSE40435 cohort **(D)**. **(E)** Univariate Cox regression analysis and identified prognostic cuproptosis regulators in TCGA-ccRCC cohort. **(F)** Venn diagram of above analysis revealed PDHB was the essential regulators among ccRCC patients.

To further identify the essential cuproptosis regulators in ccRCC, we combined expression and prognostic analysis to detect these candidates (**Figure 3A**). We gathered four differently expressed cuproptosis regulators (FDX1, PDHB, PDHA1 and CDKN2A) among TCGA-KIRC cohort and two regulators among GSE40435 cohort (**Figures 3C, D**). Meanwhile, the prognostic value of these cuproptosis-related genes was calculated by the ROC curve, which illustrated the AUC value of CDKN2A was 0.991 (95%CI: 0.982-1.000); FDX1 was 0.965 (95%CI: 0.946-0.983), and the AUC value of PDHB was 0.956 (95%CI: 0.933-0.979) (**Figure 3B**). Next, we conducted univariate Cox regression analysis and identified total 9 prognostic genes (**Figure 3E**). Taken together, the multi-omics analyses confirmed that PDHB might be the key gene involved in copper-induced cell death. (**Figure 3F**).

### PDHB is significantly downregulated in ccRCC patients

**Figure 4:**
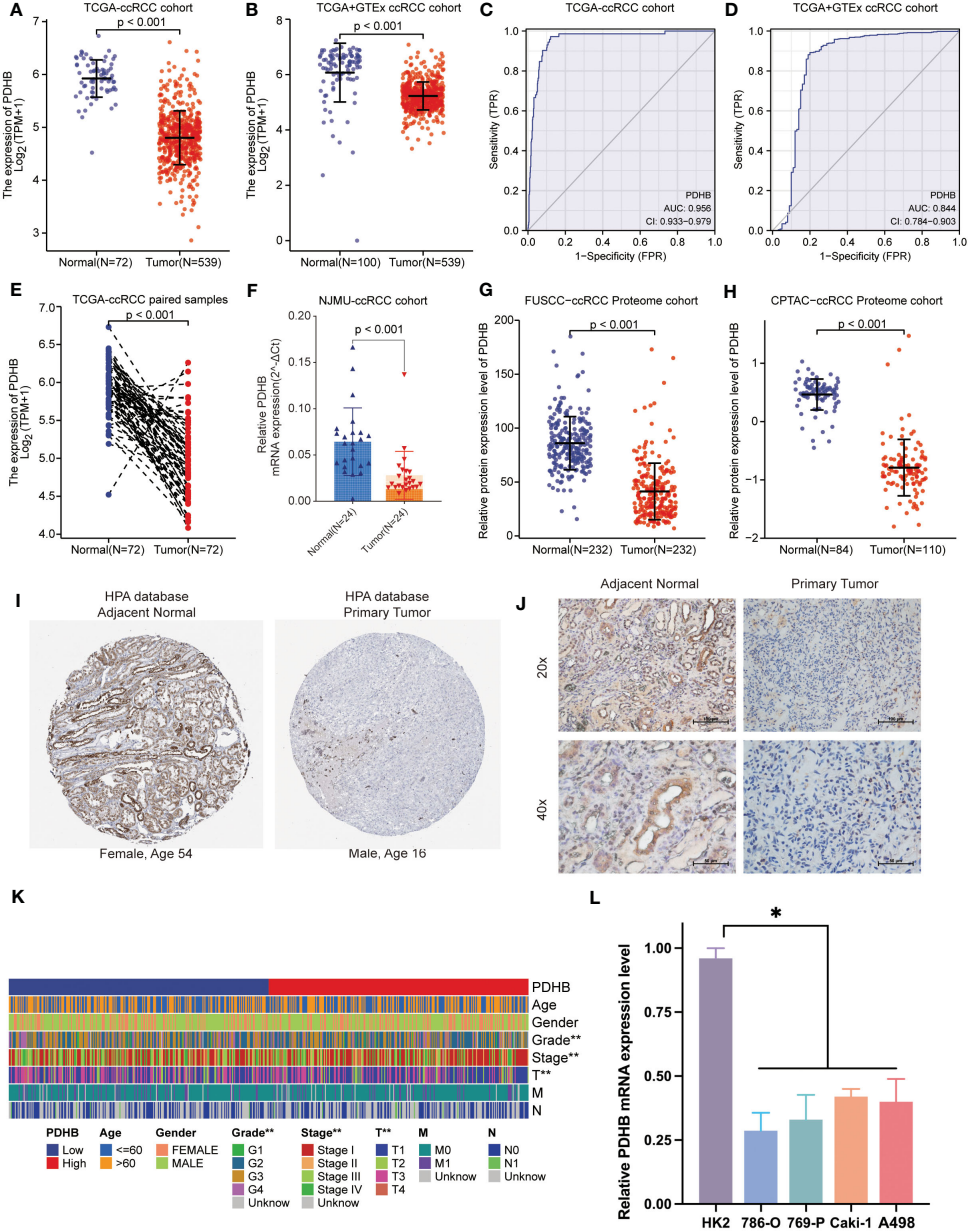
The expression pattern of PDHB in ccRCC tissues and clinical characteristics. **(A)** The expression level of the PDHB in distinct tumors or specific tumor subtypes. **(B)** The expression level of PDHB in TCGA-ccRCC and GTEx database. **(C, D)** ROC curve showed the efficiency of PDHB to distinguishing ccRCC tissue from normal tissue in TCGA **(C)** and TCGA+GTEx cohort **(D) (E)** The expression level of PDHB in TCGA-ccRCC paired samples. **(F)** The expression level of PDHB in our NJMU-ccRCC clinical samples. **(G, H)** The protein expression level of PDHB among FUSCC-ccRCC and CPTAC-ccRCC proteome cohorts. **(I)** IHC staining of ccRCC samples in HPA database. **(J)** IHC staining of our clinical ccRCC samples confirmed the down-regulated expression level in ccRCC samples compared with normal renal samples. **(K)** The correlation between expression level of PDHB and different clinicopathologic characteristics. **(L)** qRT-PCR experiments to detect the expression level of PDHB in ccRCC or normal kidney cell lines. *: p<0.05; **: p<0.01; ***: p<0.001; ns, no significant.

We first analyzed the transcriptional profiles of PDHB in ccRCC through TCGA and GTEx databases. PDHB expression was significantly lower in ccRCC tumor tissues compared with normal kidney tissues both in TCGA-ccRCC and TCGA+GTEx ccRCC cohorts (*P*<0.001; **Figures 4A, B**). This result was also validated in both TCGA paired samples and (*P*<0.001; **Figure 4E**). ccRCC samples of our NJMU cohorts, (**Figure 4F**). The ROC curve was also applied to assess the prognostic of PDHB. The AUC value of PDHB in TCGA-ccRCC cohort was 0.956 (95% CI: 0.933-0.979) and 0.844 (95% CI: 0.784-0.903) in TGCA+GTEx database (**Figures 4C, D**). Moreover, PDHB protein expression level was significantly downregulated in ccRCC tissues from CPTAC and Chinese FUSCC cohort (**Figures 4G,H**). Ultimately, its tissue abundance was measured using IHC both in HPA database and our clinical samples, which achieved consistent results from above achieved (**Figures 4I, J**). We also evaluated the association between PDHB expression and clinicopathological features. As shown in **Figure 4K**, the expression of PDHB in patients with lower stage (Stage I-II) was found significantly higher compared to patients who were highe stage (Stage III-IV) level (*P*<0.05; **Figure S2A**). The distribution of PDHB showed a significant difference among the T classification. PDHB was highly expressed in T1-2 patient compared with T3-4 patient (*P*<0.05; **Figure S2B**). Similarly, PDHB was decreased with advanced M classification (**Figure S2C**). We also performed qRT-PCR experiments to detect the expression level of PDHB in ccRCC or normal kidney cell lines and found that PDHB was down-regulated in ccRCC cell lines (*P*<0.05; 786-O, 769-P, Caki-1 and A498) compared with normal kidney cell line HK2 (**Figure 4L**). Additionally, about the subsequent analyses, median cut was used to dichotomize 539 individuals into high-PDHB (n=270) and low-PDHB (n=269) subgroup based on mRNA expression level. As shown in **Table 1**, PDHB was significantly correlated with the pathologic stage and T classification (*P*<0.001). Furthermore, logistic regression analysis was adopted to describe the exact correlativity between PDHB expression and clinicopathological characteristics (**Table 2**). Taken together, above results suggested that PDHB played a vital role in ccRCC.

**Table 1:** Baseline information of clinicopathology characteristics and PDHB expression level among TCGA-ccRCC cohort.

Characteristic	Low expression of PDHB	High expression of PDHB	*P*-value
Total	269	270	
Age, n (%)			0.413
<=60	129 (23.9%)	140 (26%)	
>60	140 (26%)	130 (24.1%)	
Gender, n (%)			0.184
Female	85 (15.8%)	101 (18.7%)	
Male	184 (34.1%)	169 (31.4%)	
Histologic grade, n (%)			0.057
G1	5 (0.9%)	9 (1.7%)	
G2	105 (19.8%)	130 (24.5%)	
G3	114 (21.5%)	93 (17.5%)	
G4	43 (8.1%)	32 (6%)	
Pathologic stage, n (%)			< 0.001
Stage I	113 (21.1%)	159 (29.7%)	
Stage II	27 (5%)	32 (6%)	
Stage III	79 (14.7%)	44 (8.2%)	
Stage IV	49 (9.1%)	33 (6.2%)	
T stage, n (%)			< 0.001
T1	116 (21.5%)	162 (30.1%)	
T2	34 (6.3%)	37 (6.9%)	
T3	114 (21.2%)	65 (12.1%)	
T4	5 (0.9%)	6 (1.1%)	
N stage, n (%)			0.809
N0	120 (46.7%)	121 (47.1%)	
N1	9 (3.5%)	7 (2.7%)	
M stage, n (%)			0.083
M0	209 (41.3%)	219 (43.3%)	
M1	47 (9.3%)	31 (6.1%)	

**Table 2:** Logistic regression analysis of PDHB and clinical information among TCGA-ccRCC cohort.

Clinical Characteristics	Total (N)	Odds Ratio (OR)	*P*-value
T stage (T2-4 vs. T1)	539	0.505 (0.358-0.711)	<0.001
N stage (N1 vs. N0)	257	0.771 (0.268-2.136)	0.618
M stage (M1 vs. M0)	506	0.629 (0.382-1.024)	0.065
Pathologic stage (Stage III-IV vs. Stage I-II)	536	0.441 (0.308-0.629)	<0.001
Age (>60 vs. <=60)	539	0.856 (0.610-1.199)	0.366
Gender (Male vs. Female)	539	0.773 (0.541-1.103)	0.156
Histologic grade (G3-4 vs. G1-2)	531	0.630 (0.446-0.887)	0.008

### Low expression of PDHB revealed unfavorable survival outcomes

**Figure 5:**
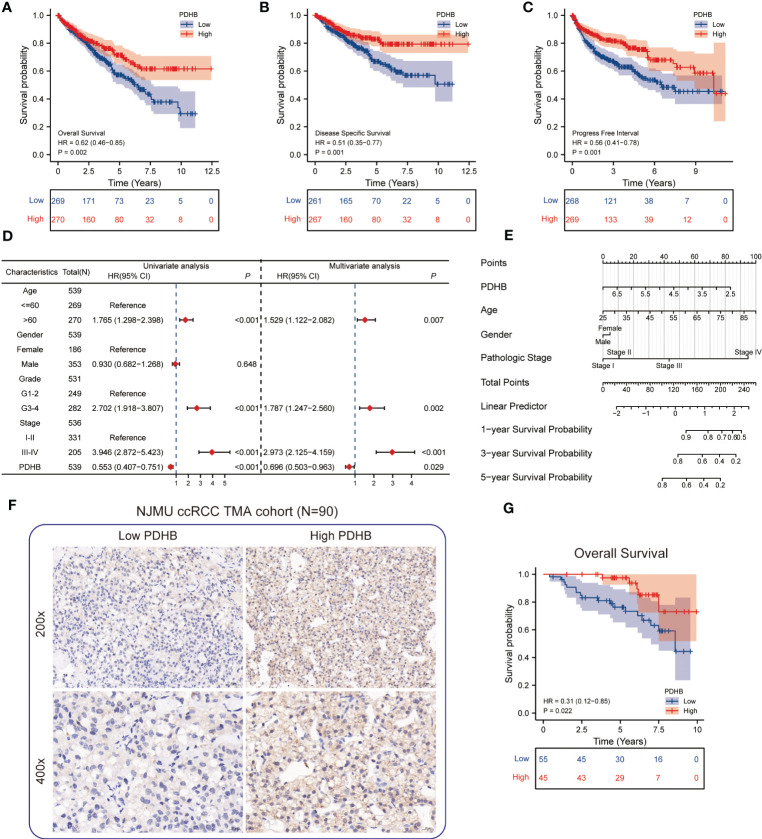
Survival analysis of PDHB and construction of nomogram **(A-C)** Kaplan–Meier curve showed the prognostic value of HNRNPC in OS, DSS, and PFI. **(D)** Univariate and multivariate Cox regression analysis in overall survival (OS). **(E)** Construction of a nomogram for estimation of survival rates for ccRCC patients. **(F)** Representative images of IHC staining of low and high PDHB expression in tissue microarray (N=90). **(G)** Kaplan–Meier survival analysis revealed low expression of PDHB revealed unfavorable clinical outcomes among our NJMU-ccRCC cohort.

From TCGA-ccRCC database, we found that the patients with low level of PDHB displayed poor prognosis in overall survival (OS), disease-specific survival (DSS) and progression free interval (PFI) (**Figures 5A–C**). In addition, conducting univariate and multivariate cox regression, PDHB could serve as an independent predictive marker for ccRCC patients’ overall survival (Univariate: HR=0.553, 95% CI=0.407−0.751, *P*<0.001; Multivariate: HR=0.696, 95% CI=0.503-0.963, *P*=0.029), revealing that low levels of PDHB expression were correlated with shorter OS (**Figure 5D**). Moreover, a nomogram based on age, gender, pathologic stage, and PDHB was developed to predict the 1-, 3-, and 5-year OS for individual ccRCC patients. (**Figure 5E**). Additionally, by analyzing PDHB expression from IHC tissue microarray staining from our NJMU ccRCC cohort (N=90), we divided patients into PDHB-high and PDHB-low subgroups (**Figure 5F**). Kaplan-Meier survival curves demonstrated that low expression of PDHB were correlated with shorter overall survival (*P*=0.022; **Figure 5G**). Therefore, these findings suggested that PDHB might serve as an indicator for the clinical prognosis of ccRCC patients.

### Functional enrichment and pathway annotation of PDHB

**Figure 6:**
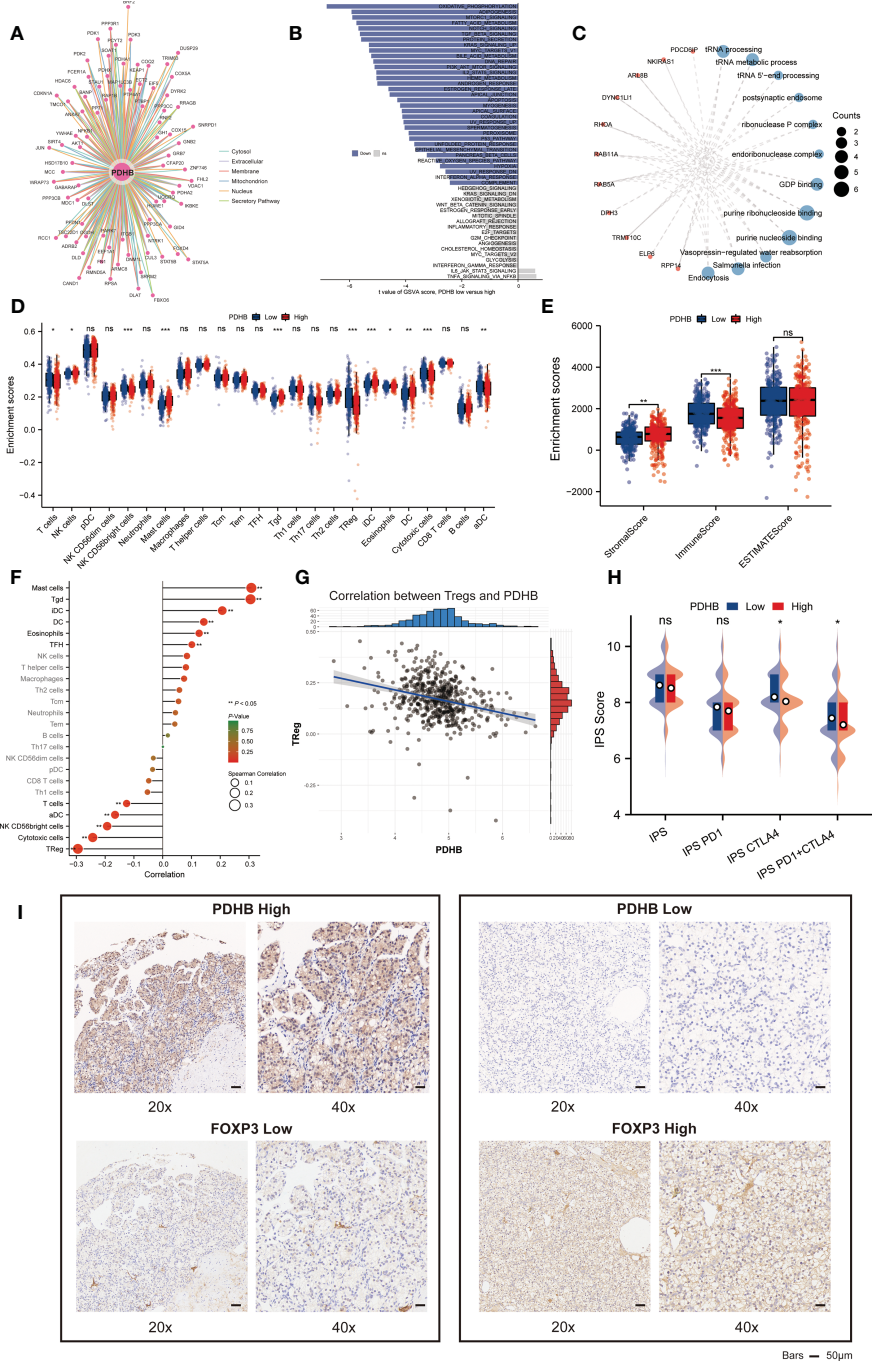
Functional enrichment analysis and Relationship of immune infiltration among tumor microenvironments. **(A)** ComPPI database for constructing a cellular compartment-specific protein-protein interaction network of PDHB. **(B)** GSVA analysis illustrated that PDHB participated in several Hallmark pathways. **(C)** GO and KEGG pathway enrichment of PDHB related genes. **(D-F)** the difference and correlation between PDHB expression and various immune cells and ESTIMATE score. **(G)** The correlation analysis between PDHB expression and Treg cells. **(H)** Assessment of IPS score among different PDHB expression level patients **(I)** IHC experiments among NJMU-ccRCC tissue microarray cohort to detect the expression level of Treg cell marker FOXP3. *: p<0.05; **: p<0.01; ***: p<0.001; ns, no significant.

PPI network was constructed and illustrated in ComPPI database (**Figure 6A**). We next investigated the difference among biological function, hallmarks and pathways involved. GSVA analysis demonstrated that oxidative phosphorylation, adipogenesis, mTORC1 signaling and fatty acid metabolism pathway was significantly enriched in PDHB low subgroup (**Figure 6B**). GSEA analysis also acquired similar enrichment of Hallmark bile acid metabolism and apical surface signature in PDHB-low subgroup (**Figures 6C**, **S3A, B**). By calculating PDHB co-expressed genes (**Figures S3A, B**), we found that co-expressed genes were involved in tRNA processing and GDP binding pathway. These findings confirmed that down-regulated PDHB was mainly participated in metabolism-related pathway.

### Correlation between PDHB and characteristics of tumor microenvironment

We applied ssGSEA algorithm to evaluate 24 types of immune cell infiltration level among tumor microenvironment (TME). Detailed characteristics of immune cell proportion and differences between PDHB expression subgroup was further identified. The results revealed that regulatory T cells (Treg) cells were dramatically increased in low-PDHB patients, which demonstrated a suppressive tumor immune microenvironment (**Figure 6D**). Then, we investigated the component of immune cell and stromal cell using ESTIMATE algorithm. As shown in **Figure 6E**, high-PDHB expression subgroup tend to illustrate more immune and stromal cell infiltration phenomenon. Additionally, correlation analysis indicated that Treg, cytotoxic cells, NK CD56bright cells and T cells was negatively correlated with PDHB expression level, while mast cells, Tgd, DC, eosinophils and Tfh cells has a significant positive correlation with PDHB (**Figure 6F**). Additionally, we found negative association between the expression level of PDHB and Treg cell marker FOXP3 in TCGA-ccRCC cohort. Ultimately, we performed IHC assays to detect the expression level of PDHB and Treg cell marker FOXP3, which found a negative correlation between PDHB and Treg cells in our NJMU ccRCC cohort (**Figure 6I**). Above results suggested that low expression of PDHB plays a vital role in regulating suppressive tumor immune microenvironment mainly *via* up-regulating Treg cells by ssGSEA and CIBERSOFT algorithms (**Figure 6G**). We assessed the IPS score among different PDHB expression level patients, which could predict the response to immunotherapy. Among them, IPS score of CTLA-4 block therapy and CTLA4+PD-1 combined block therapy was significantly increased in high-PDHB group (**Figure 6H**). These results illustrated low-PDHB expression patients are more likely to benefit from CTLA-4 block therapy and CTLA4+PD-1 combined block immunotherapy.

### Knockdown PDHB promoted proliferation and migration of ccRCC *in vitro* and *in vivo*

**Figure 7:**
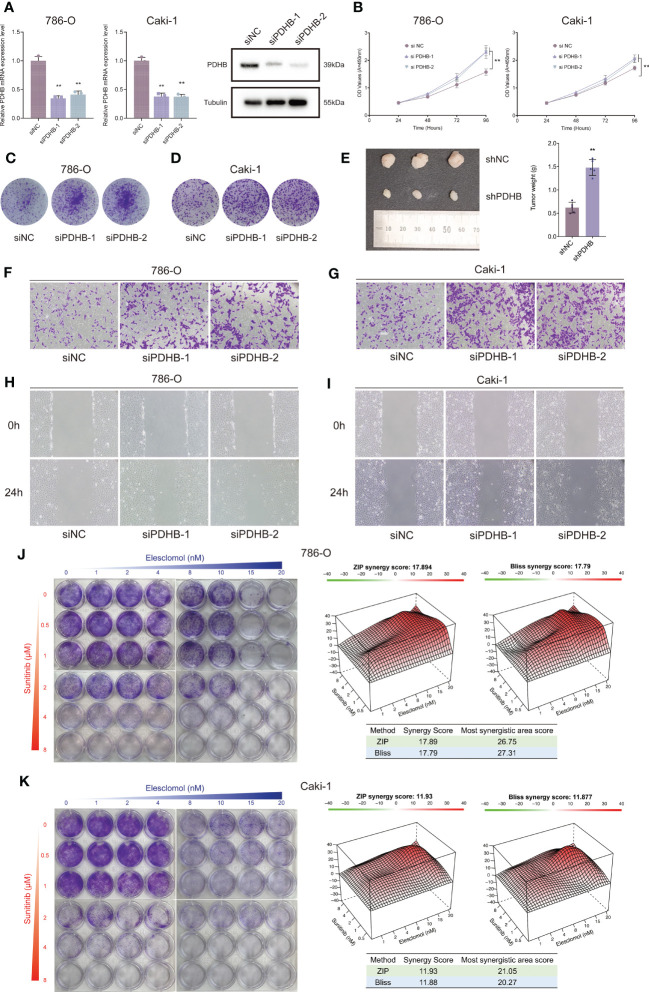
Silencing essential cuproptosis regulator PDHB promoted ccRCC proliferation, migration and sunitinib resistance. **(A)** qRT-PCR to confirm the performance of siRNA targeting PDHB among 786-O and Caki-1 RCC cells. **(B)** CCK-8 assay results indicated that PDHB knockdown increased cell proliferation. **(C, D)** Colony-formation efficiency of knockdown PDHB in 786-O and Caki-1 cells. **(E)** Knocking down PDHB dramatically accelerated tumor growth *in vivo*. **(F, G)** Transwell migration assay of knockdown PDHB and control group. **(H, I)** Wound healing assay of knockdown PDHB and control group. **(J, K)** Colony formation assays for the assessment of inhibition rate 786-O and Caki-1 cells treated with sunitinib and elesclomol for 10 days, while the synergy score plot based on ZIP and Bliss models.

To further determine the biological oncogenic role of PDHB in ccRCC, PDHB-was knocked down in 786-O and Caki-1 cell models and validated by qRT-PCR and western blotting (**Figure 7A**, *P*< 0.05). Cell counting kit-8 (CCK-8) assay indicated that PDHB knockdown significantly increased cell proliferation ability (**Figure 7B**). Colony formation assay was also employed to determine the long-term impact of PDHB on cells proliferation. We observed higher colony-formation efficiency in PDHB knockdown group than control group both in 786-O and Caki-1 cell lines accordingly (**Figures 7C, D**). Ultimately, *in vivo* experiment confirmed that knocking down PDHB dramatically accelerated tumor growth (**Figure 7E**). In addition, Transwell migration assay and wound healing assay demonstrated that knockdown PDHB increased the migration ability of RCC cells (**Figures 7F–I**). These findings corroborated that PDHB was essential for ccRCC proliferation and metastasis *in vitro* and *in vivo*.

### Copper-induced cell death activation overcomes sunitinib resistance in ccRCC cells

Currently, sunitinib is a first line recommended clinical treatment drug that targets multiple RTKs, such as VEGFR2 (Flk-1) and PDGFRβ (51, 52). Considering that sunitinib resistance is still a common challenge for targeted therapies among renal cell carcinoma (53), we sought to investigate the combined therapeutic strategies to overcome sunitinib resistance in RCC cells. Importantly, the combination therapy of elesclomol and sunitinib profoundly suppressed the proliferation ability of ccRCC cells in a synergistic manner, as demonstrated by the HSA and Bliss synergy scores (786-O: ZIP-score: 17.89, Bliss-score: 17.79; Caki-1: ZIP-score: 11.93, Bliss-score: 11.88; **Figures 7J, K**). Ultimately, among the most synergistic area score also demonstrated that elesclomol and sunitinib could suppress ccRCC cells proliferation synergistically (Most synergistic area score: 786-O: ZIP-score: 26.75, Bliss-score: 27.31; Caki-1: ZIP-score: 21.05, Bliss-score: 20.27; **Figures 7J, K**).

## Discussion

Clear cell renal cell carcinoma is the most common and lethal histological subtype of RCC (2, 54). About 15% of RCC patients are metastatic while detected (55). Interest in investigating the possible targeting of particular immune-related biomarkers for immunotherapy has increased as a result of the efficacy of immune checkpoint inhibitors in treating ccRCC (56). Nevertheless, there are currently no clinically applicable markers to assess heterogeneous molecular subgroups and reliably predict their prognostic outcome in clinic treatment (4, 57, 58).

Recently, accumulating studies revealed that intracellular copper (Cu) induces a novel form of regulated cell death that is different from oxidative stress-related cell death (apoptosis, ferroptosis, and necroptosis), which has been termed “cuproptosis” (22–25). Understanding how cuproptosis is initiated, propagated, and ultimately executed may presented a new perspective on therapeutic interventions and possible combination treatments (59–61). However, the role and underlying mechanism of cooper-induced cell death in ccRCC remained unclear. To determine the specific regulator of copper-mediated cytotoxicity in ccRCC, we first obtained ten essential cuproptosis regulators by genome-wide CRISPR-Cas9 loss-of-function screens. Then, combined with multi-omics analysis, PDHB was selected as the essential cooper-induced cell death regulator.

Localized in the mitochondria, pyruvate dehydrogenase B (PDHB) is the enzyme that catalyzes the glucose-derived pyruvate to the acetyl-CoA and plays important role in oxidative phosphorylation (62). Zhu et al. showed that miR-146b-5p could regulate colorectal cancer proliferation, invasion and glycolysis directly targeting PDHB (63). Similarly, other researchers found that PDHB was involved in circadian clock and could regulates metabolic phenotype in colorectal cancer, which influenced tumor progression and drug response (64). However, the mechanism and biological function of PDHB in ccRCC is still poorly understood.

Our study first illustrated the landscape of dysregulation of cuproptosis regulators across human cancer and found distinct expression pattern of cuproptosis regulators in ccRCC. In order to discover the most important cuproptosis regulators in ccRCC, we performed multi-omics screens and confirmed PDHB as an essential component regulating in ccRCC progression. qRT-PCR and IHC was further validated in our NJMU-ccRCC cohort. Moreover, our research revealed high PDHB expression level was associated with favorable survival outcomes in both TCGA database and our clinical cohort. Functional enrichment and pathway annotation demonstrated that PDHB was involved in oxidative phosphorylation and fatty acid metabolism pathway, which was corresponding with the feature of cuproptosis: lipoylated TCA cycle proteins-mediated novel cell death pathway (65, 66). Furthermore, cuproptosis-related gene PDHB might inhibit the progression of ccRCC by mediating immune-active tumor microenvironment associated with cell death and immune responses.

Ultimately, we analyzed the correlation between immune characteristics among tumor microenvironment and PDHB expression level. Our finding revealed Treg, cytotoxic cells, NK CD56bright cells and T cells was negatively correlated with PDHB, indicating low PDHB may contribute immune suppressive microenvironment. Recent research provided that Tregs are one mechanism of tumor-driven immune evasion that provide prototypical targets for testing novel anticancer strategies within the newer paradigm (67). The dysfunction of Tregs may contributed to immune dysfunction, immune suppression and sunitinib resistance (68–70). Based on above evidence, we provided an innovative combination strategy for treating ccRCC populations. Our findings demonstrated that copper-induced cell death activation overcomes sunitinib resistance in ccRCC cells. However, the mechanisms underlying PDHB’s tumorigenic actions are still not entirely clarified. Before we can target this protein in patients safely and effectively, further research is required to describe the comprehensive molecular mechanisms of PDHB.

## Conclusions

In summary, our research illustrated the dysregulation of cuproptosis regulators across human cancer and revealed its expression pattern, survival outcomes and biological function n ccRCC. As a hub cuprotosis-related regulators, low PDHB expression closely associated with immune suppressive microenvironment and sunitinib resistance, which mainly *via* regulating Tregs. Therefore, PDHB could serve as a potential prognostic biomarker and immune-regulation factor for ccRCC.

## Data availability statement

The original contributions presented in the study are included in the article/**Supplementary Material**. Further inquiries can be directed to the corresponding authors.

## Ethics statement

The studies involving human participants and clinical samples were approved by the First Affiliated Hospital of Nanjing Medical University (No. 2021-SR-430). The patients provided their written informed consent to participate in this study.

## Author contributions

CM and XW conceived of the study and carried out its design. JW, YL and SW performed experiments and wrote the paper. CM and SW revised the paper. All authors read and approved the final version of this manuscript. All authors agreed to be accountable for all aspects of the work.

## Supplementary material

Supplementary Figure 1 Single-cell RNA-seq quality control and pretreatment. **(A)** “Harmony” and UMAP algorithm to remove batch effects and gathered a total of 33 clusters. **(B)** Detailed cell annotation of single-cell RNA-seq. **(C)** Dotplot illustrated the marker genes between each cluster.

Supplementary Figure 2 The expression level of PDHB and clinical characteristics. **(A-C)** The expression level of PDHB was analyzed by different clinicopathologic characteristics. **(A)** Stage I-II versus Stage III-IV. **(B)** T1-T2 versus T3-T4. **(C)**. M0 versus M1.

Supplementary Figure 3 GSEA analysis and functional enrichment of PDHB. **(A)** GSEA analysis enrichment demonstrated that Hallmark bile acid metabolism and apical surface signature in PDHB-low subgroup. **(B)** GSEA analysis illustrated that PDHB participated in several KEGG metabolism-related pathways. **(C)** Heatmap showed the co-expressed genes of PDHB.

Supplementary Figure 4 Correlation analysis between PDHB and Treg cells. **(A)** Correlation analysis between PDHB and Treg cells by “CIBERSORT” algorithm. **(B)** Correlation analysis between PDHB and Treg cell marker gene FOXP3 in TCGA-ccRCC cohort.
