# Supplementary figures and images for: Integrated single-cell and bulk characterization of cuproptosis key regulator PDHB and association with tumor microenvironment infiltration in clear cell renal cell carcinoma
Source: Front Immunol. 2023 Jun 7;14:1132661. doi: 10.3389/fimmu.2023.1132661 (PMC10282190; doi:10.3389/fimmu.2023.1132661)

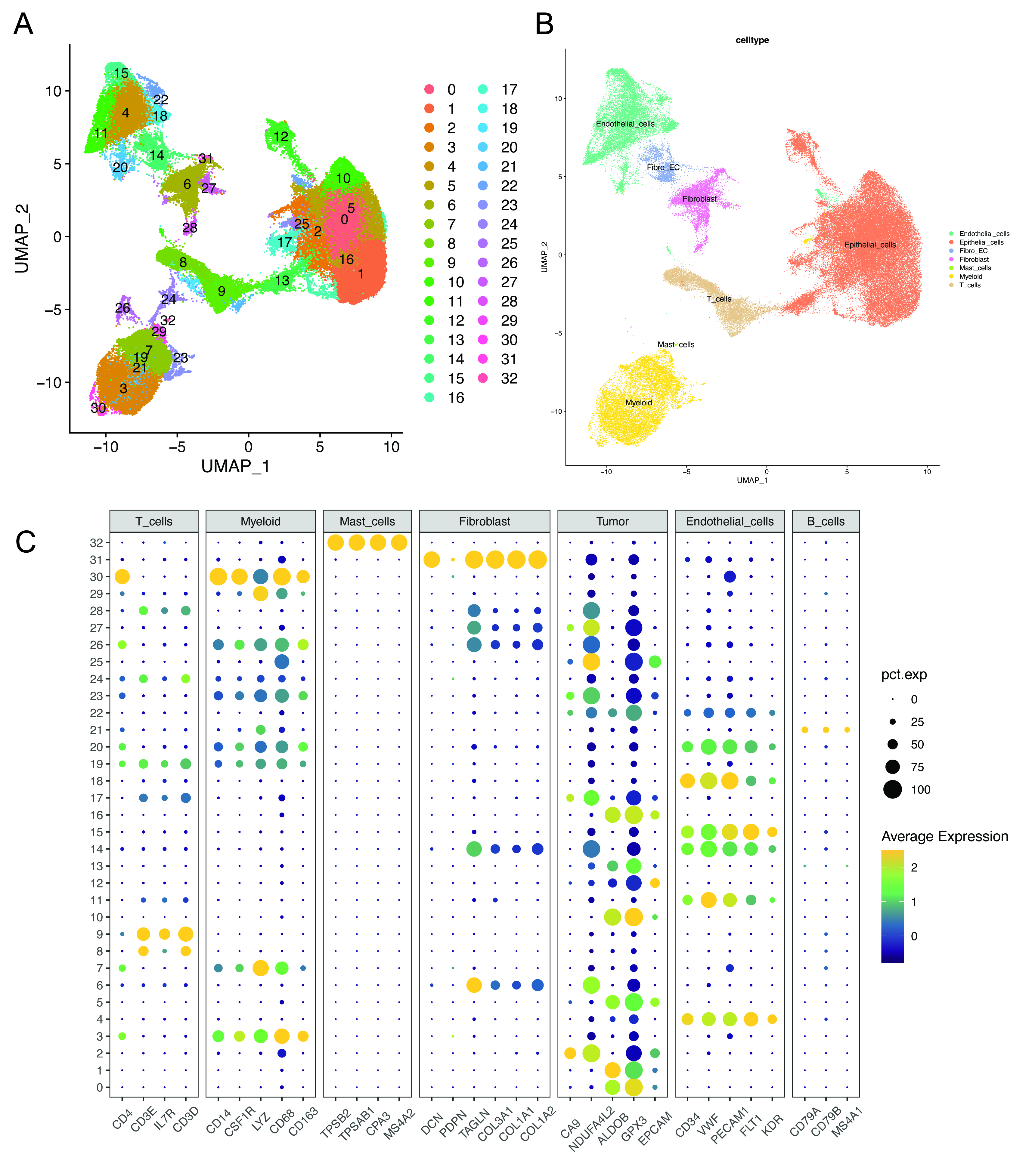

Supplement: Supplementary Figure 1 — Single-cell RNA-seq quality control and pretreatment. (A) “Harmony” and UMAP algorithm to remove batch effects and gathered a total of 33 clusters. (B) Detailed cell annotation of single-cell RNA-seq. (C) Dotplot illustrated the marker genes between each cluster. [file Image_1.jpeg]

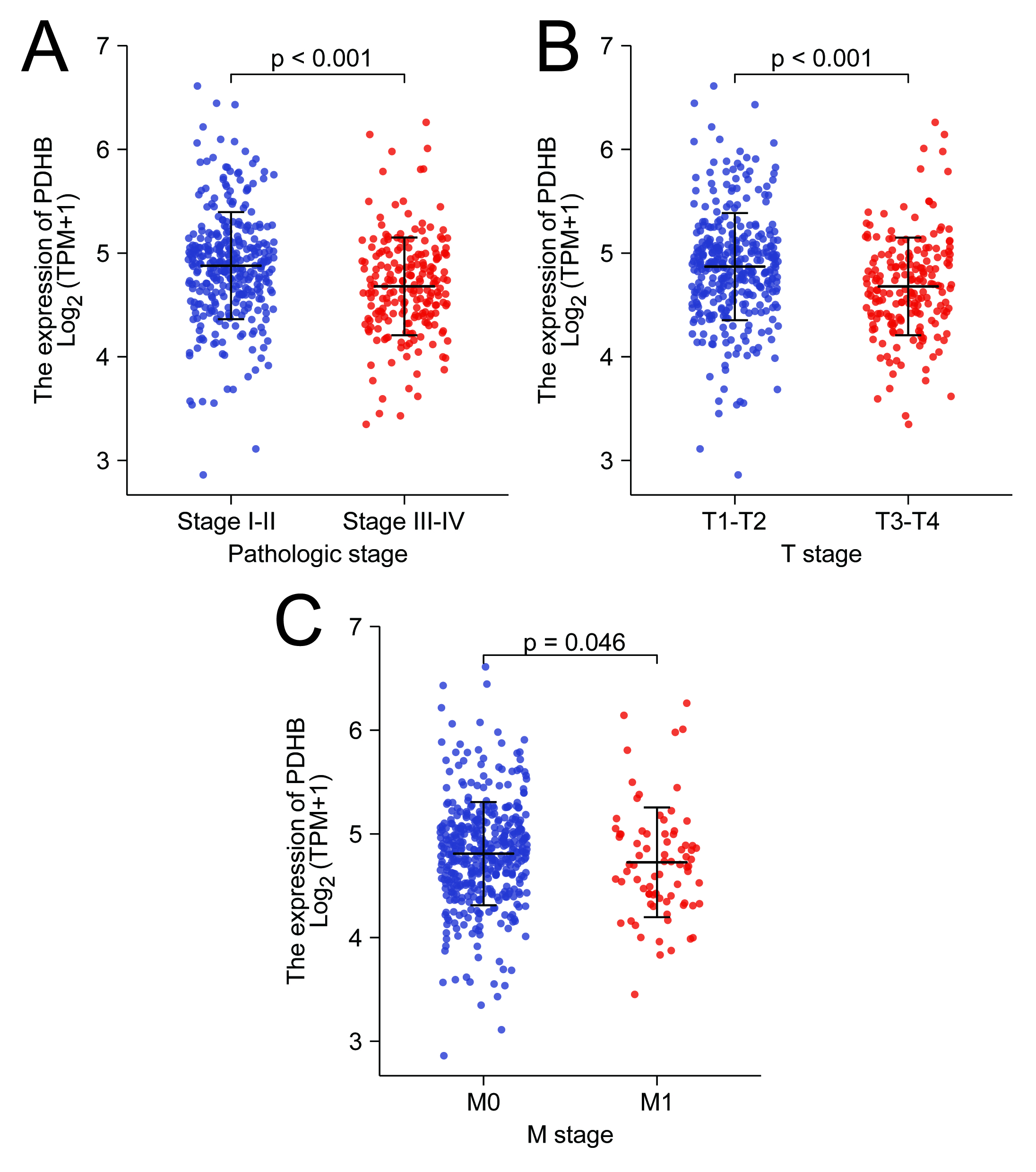

Supplement: Supplementary Figure 2 — The expression level of PDHB and clinical characteristics. (A-C) The expression level of PDHB was analyzed by different clinicopathologic characteristics. (A) Stage I-II versus Stage III-IV. (B) T1-T2 versus T3-T4. (C). M0 versus M1. [file Image_2.jpeg]

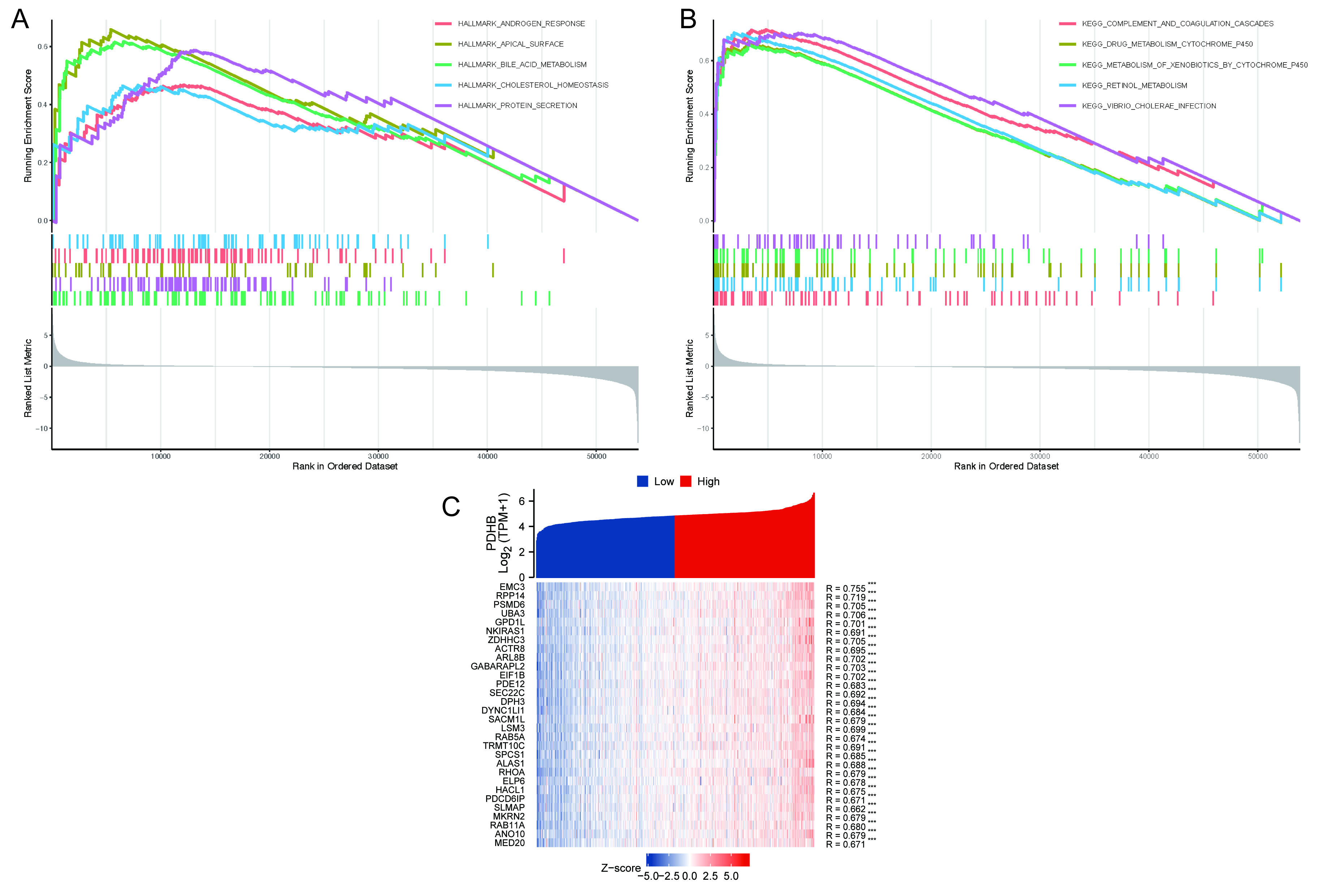

Supplement: Supplementary Figure 3 — GSEA analysis and functional enrichment of PDHB. (A) GSEA analysis enrichment demonstrated that Hallmark bile acid metabolism and apical surface signature in PDHB-low subgroup. (B) GSEA analysis illustrated that PDHB participated in several KEGG metabolism-related pathways. (C) Heatmap showed the co-expressed genes of PDHB. [file Image_3.jpeg]

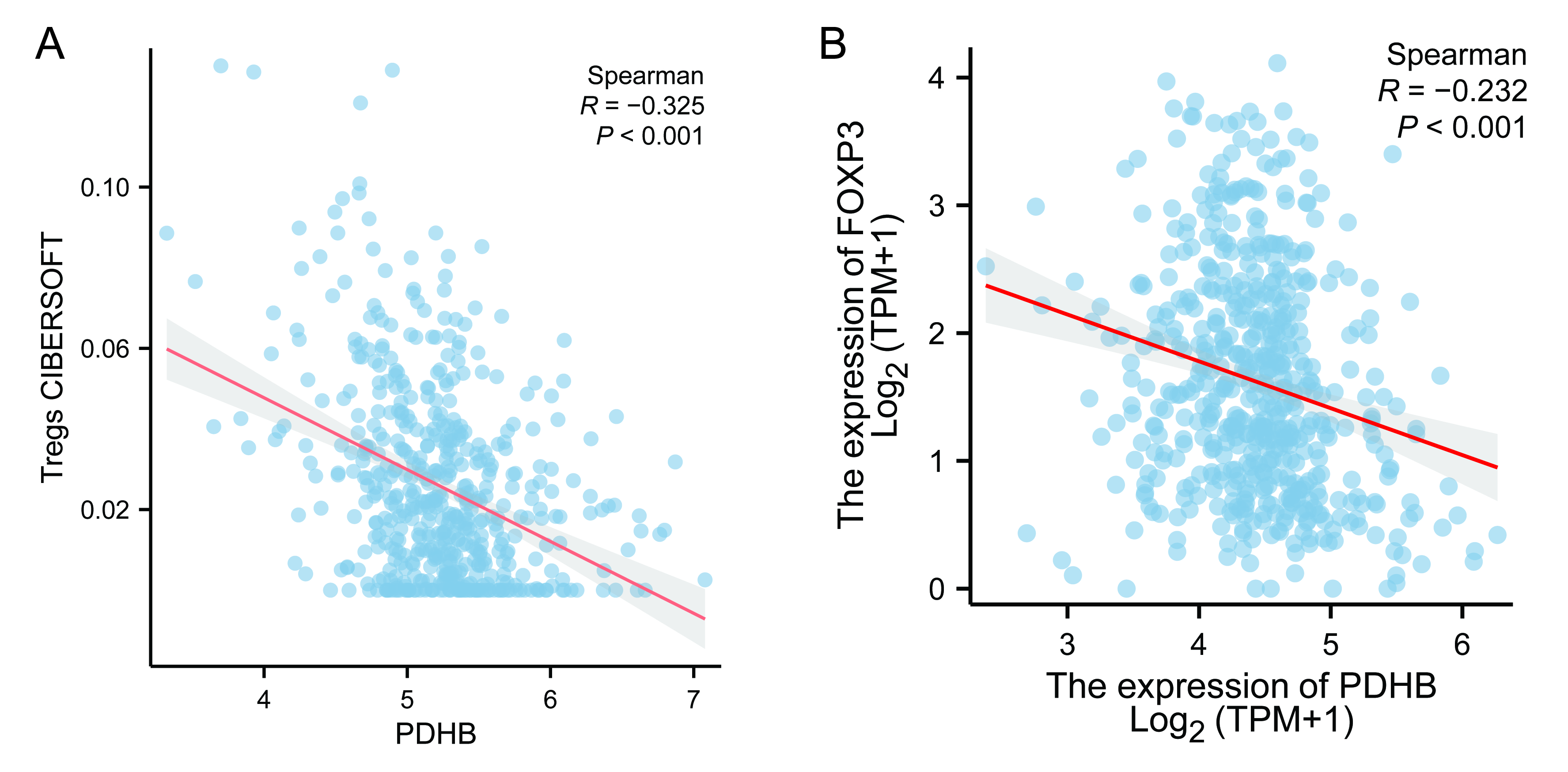

Supplement: Supplementary Figure 4 — Correlation analysis between PDHB and Treg cells. (A) Correlation analysis between PDHB and Treg cells by “CIBERSORT” algorithm. (B) Correlation analysis between PDHB and Treg cell marker gene FOXP3 in TCGA-ccRCC cohort. [file Image_4.jpeg]
