# Supplementary material for: Integrated single-cell and bulk characterization of cuproptosis key regulator PDHB and association with tumor microenvironment infiltration in clear cell renal cell carcinoma
Source: Front Immunol. 2023 Jun 7;14:1132661. doi: 10.3389/fimmu.2023.1132661 (PMC10282190; doi:10.3389/fimmu.2023.1132661)
Supplement: Supplementary file 5 [file Table_1.docx]

**Table S1 Oligonucleotide sequences used in this research**

| Primers | Sequences | |
| --- | --- | --- |
| PDHB | Forward | AAGAGGCGCTTTCACTGGAC |
|  | Reverse | ACTAACCTTGTATGCCCCATCA |
| β-actin | Forward | ATGACTTAGTTGCGTTACACC |
|  | Reverse | GACTTCCTGTAACAACGCATC |
| siRNA-NC | Forward | UUCUCCGAACGUGUCACGUTT |
|  | Reverse | ACGUGACACGUUCGGAGAATT |
| siRNA-PDHB-1 | Forward | CAAAGAUUCUAGAGGACAATT |
|  | Reverse | UUGUCCUCUAGAAUCUUUGTT |
| siRNA-PDHB-2 | Forward | GACAGUUCGUGAUGCUAUATT |
|  | Reverse | UAUAGCAUCACGAACUGUCTT |
